# Relationship Between Vitamin D Status and Vitamin D Receptor Gene Polymorphisms With Markers of Metabolic Syndrome Among Adults

**DOI:** 10.3389/fendo.2018.00448

**Published:** 2018-08-16

**Authors:** Tatiana Karonova, Elena Grineva, Olga Belyaeva, Anna Bystrova, Edward B. Jude, Alena Andreeva, Anna Kostareva, Pawel Pludowski

**Affiliations:** ^1^Institute of Endocrinology, Almazov National Medical Research Centre, St. Petersburg, Russia; ^2^Internal Medicine Department, Pavlov First Saint Petersburg State Medical University, St. Petersburg, Russia; ^3^Tameside Hospital NHS Foundation Trust, Ashton Under Lyne, United Kingdom; ^4^Institute of Molecular Biology and Genetics, Almazov National Medical Research Centre, St. Petersburg, Russia; ^5^Department of Biochemistry, Radioimmunology and Experimental Medicine, The Children's Memorial Health Institute, Warsaw, Poland

**Keywords:** metabolic syndrome, obesity, diabetes, dyslipidemia, VDR gene polymorphisms

## Abstract

**Introduction:** Recent studies have demonstrated that vitamin D deficiency contributes to the development of metabolic disorders, including obesity and type 2 diabetes mellitus (T2DM). Several vitamin D receptor (VDR) gene polymorphisms had been described to play a role in these conditions since vitamin D receptors were found in many tissues. The aim of this study was to assess the relationship between vitamin D status and VDR gene polymorphisms with metabolic syndrome (MS) parameters in Russian middle-aged women.

**Materials and Methods:** A total of 697 women aged between 30 to 55 years were included in this cross-sectional study. Serum 25-hydroxyvitamin D (25(OH)D) level and four VDR gene polymorphisms rs1544410 (*BsmI*), rs7975232 (*ApaI*), rs731236 (*TaqI*), and rs2228570 (*FokI*) were measured. We applied the International Diabetes Federation (IDF) criteria to identify subjects with MS.

**Results:** 9.3% of subjects had normal vitamin D level, while 90.7% were insufficient or deficient. Abdominal obesity (AO) was seen in 75.5%, impaired glucose tolerance (IGT) or T2DM was observed in 33.3%, reduced high-density lipoprotein cholesterol (HDL-C) level in 32.2% and hypertriglyceridemia in 23.4%. Serum 25(OH)D level in women with or without MS did not differ (48.6 ± 1.8 and 51.1 ± 1.5 nmol/l, *p* > 0.05). Subjects with vitamin D deficiency showed an increased risk of AO [CI 95% 2.23; 1.15–4.30] and low HDL-C [CI95% 2.60; 1.04–6.49] compared to subjects with normal 25(OH)D level. IGT and T2DM risk was increased only when 25(OH)D concentration was less than 39.0 nmol/l [CI 95% 7.17; 2.99–17.7], but risk of MS did not differ in normal vitamin D status subjects and insufficient/deficient ones (*p* > 0.05). T allele carriers (*A*) of rs7975232 had higher total cholesterol and low-density lipoprotein cholesterol levels compared with the GG (*aa*) genotypes. Similarly, GG (*BB)* genotype carriers of rs1544410 had higher triglyceride levels than subjects with A (*b)* allele carriers. However VDR gene polymorphisms did not seem to be associated with an increased risk of MS.

**Conclusions:** Vitamin D deficiency, rs7975232, and rs1544410 VDR gene variants are associated with MS parameters in Russian middle-aged women.

## Introduction

It is well known that obesity, impaired glucose tolerance (IGT) or diabetes mellitus type 2 (T2DM), hypertension, high low-density lipoprotein cholesterol (LDL-C), hypertriglycedemia and decreased high-density lipoprotein cholesterol (HDL-C) are associated with high risk of cardiovascular disease and mortality (1–5). Different combinations of these factors have been defined as “Metabolic Syndrome” (MS). The International Diabetes Federation (IDF) defines MS as abdominal obesity (waist circumference ≥94 cm for males and ≥80 cm for females) plus two of the others cardiovascular risk factors: hyperglycemia, T2DM, hypertension, hypertriglyceridemia, and low HDL-C level (6). However, there are other definitions of MS, for example the World Health Organization includes insulin resistance as an essential component to define MS (7).

Interestingly, recent studies showed a possible negative effect of vitamin D deficiency in the development of metabolic disorders, including obesity, IGT, T2DM, dyslipidemia and hypertension (8–11). These relationships are not clear. Some studies demonstrated inverse associations between serum 25-hydroxyvitamin D (25(OH)D) level and MS (12–14), but others have failed to do so (15–17). Analysis of data from the National Health and Nutritional Examination Survey (NHANES) demonstrated that concentrations of 25(OH)D were significantly associated with MS prevalence among Americans (18). In addition, dose-response meta-analysis showed a relationship between serum 25(OH)D level and MS in cross-sectional but not in longitudinal studies (19).

It is not surprising that vitamin D could be associated with cardiovascular risk factors. Vitamin D is not only involved in calcium and bone metabolism, but also in the regulation of proliferation and differentiation of many cells, in addition to its immunoregulatory, antiangiogenetic, and antioxidant properties. Pleiotropic extraskeletal effects of vitamin D are mediated by the activation of vitamin D receptors (VDR) which are widely expressed in different cells (20, 21).

Several polymorphisms have been reported for the VDR gene such as rs7975232 (*ApaI*), rs1544410 (*BsmI*), rs2228570 (*FokI*), and rs731236 (*TaqI*). Recent studies demonstrated that some of these polymorphisms are associated with T2DM and insulin secretion (22) as well as with metabolic disturbances in obese people (23).

Some studies in the Russian Federation confirmed high prevalence of MS and its components in the general population (24). Studies have also demonstrated high prevalence of vitamin D deficiency and insufficiency in Russia (25). However the relationship between MS components and vitamin D status in this population has not been assessed.

The aim of this study was to find out whether there is an association between vitamin D status and VDR gene polymorphisms with MS parameters.

## Materials and methods

### Study population

We conducted a cross-sectional study of 697 Caucasian women aged between 30–55 years and residing in Saint-Petersburg, Russia. The subjects were recruited during outpatient clinic visits for minor medical problems. Exclusion criteria included clinically significant kidney and gastrointestinal diseases, history of diabetes mellitus, regular insolation (every week) and use of vitamin D supplements. The study was conducted in compliance with the principles of the Declaration of Helsinki, and each participant gave written informed consent before enrollment. The study was approved by the local ethics committee.

### Data collection

Anthropometric measurements included body weight (kg), height (cm), waist circumference (WC; cm), and body mass index (BMI) that was determined using the following formula: BMI = weight (kg)/height (m2). All measurements were performed in the morning with the participants dressed in light clothing, without shoes. Waist circumference was measured with the subjects standing and at the midpoint between the lower rib margin and the iliac crest parallel to the floor. Abdominal obesity was defined as WC ≥ 80 cm. Blood pressure was measured in the right arm in a sitting position after 10-min rest.

Blood samples were taken after an overnight fast and stored at −70°C. Serum 25(OH)D concentrations were measured by a chemiluminescent immunoassay (Abbott Architect 8000, Deerfield, IL, USA; intra-assay CV of 1.60–5.92%, inter-assay CV ranged from 2.15 to 2.63%).

Fasting plasma glucose (FPG) was determined enzymatically using commercially available kits and auto analyzer (UniCel DxC 800, Brea, CA, USA). Serum lipids: total cholesterol (TC), triglycerides (TG), HDL-C, and LDL-C were measured by enzymatic colorimetric assays with the analyzer COBAS INTEGRA 400/700/800 and standard kits (Roche Diagnostics, Mannheim, Germany).

### Genetic analysis

Genomic DNA was extracted from peripheral white blood cells using phenol extraction method. VDR gene polymorphisms rs7975232 (*ApaI*), rs1544410 (*BsmI*), rs2228570 (*FokI*), and rs731236 (*TaqI*) were detected by polymerase chain reaction-restriction fragment length polymorphism method. VDR alleles and genotypes were determined by the presence or absence of *BsmI, ApaI, TaqI*, and *FokI* restriction enzyme sites. Digestion pattern assessment indicated the following VDR genotypes: *BsmI (BB, Bb, bb), ApaI (AA, Aa, aa), TaqI (TT, Tt, tt), and FokI (FF, Ff, ff)*. VDR genotyping was performed in 454 study subjects, while 236 healthy subjects represented the control group for genetic analysis.

### Data analysis

Metabolic syndrome was diagnosed using IDF criteria: systolic blood pressure (SBP) ≥130 mmHg or diastolic blood pressure (DBP) ≥85 mmHg or treatment for hypertension; TG ≥1.7 mmol/L and HDL-C <1.29 mmol/l or treatment for dyslipidemia; plasma glucose level more than 5.6 mmol/l or IGT/T2DM during oral glucose tolerance test (6).

Vitamin D status was classified according to the Endocrine Society criteria (26). Vitamin D deficiency was defined as serum 25(OH)D level <50 nmol/l, insufficiency 50-75 nmol/l and sufficiency ≥75 nmol/l.

Statistical analyses were performed using SPSS software 17.0 for Windows (SPSS Inc, Chicago, Ill). Data were presented as a percentage or mean ± SD. Frequencies of qualitative indicators were compared by nonparametric techniques using chi square (χ^2^). Comparison of quantity indicators was performed using ANOVA. Statistical significance was defined as *p* < 0.05.

## Results

The study group included 697 Caucasian women aged 30–55 years, mean age was 43.5 ± 0.3 years. One hundred and seventy one participants (24.5%) had WC less than 80 cm whereas abdominal obesity was revealed in 526 subjects (75.5%). Thirty one patients (4.5%) were newly diagnosed with T2DM and IGT was noted in 201 participants (28.8%). One hundred and seventy five patients (25.1%) had arterial hypertension. Increased TC was observed in 342 women (49.0%), hypertriglyceridemia in 163 (23.4%), elevated LDL-C in 443 (63.6%), and reduced HDL-C in 224 (32.2%) participants.

The results demonstrated significantly higher BMI, BP, levels of fasting plasma glucose, TC, LDL-C, TG, and lower HDL-C in subjects with abdominal obesity compared to those with WC < 80 cm.

Serum 25(OH)D assessment revealed vitamin D deficiency and insufficiency in 632 women (90.7%), while only 65 participants (9.3%) had 25(OH)D concentrations ≥75 nmol/l. Vitamin D deficiency was more frequently observed in overweight and obese women (χ^2^ = 4.32, *p* < 0.05 and χ^2^ = 6.29, *p* < 0.05, respectively). Prevalence of vitamin D deficiency and insufficiency did not differ between subjects with and without abdominal obesity. The basic characteristics of study participants are presented in Table 1.

**Table 1:** Clinical and laboratory characteristics of women with and without abdominal obesity.

**Characteristics**	**Abdominal obesity** **(+)**	**Abdominal obesity** **(−)**	***P*-value**
Age, years	45.0 ± 0.3	40.4 ± 0.6	< 0.05
WC, cm	95.7 ± 12.1	72.4 ± 4.7	< 0.001
BMI, kg/m^2^	30.5 ± 5.9	22.3 ± 2.5	< 0.001
**BP, mmHg**			
Systolic	127.8 ± 17.9	118,86 ± 18.7	NS
Diastolic	80.1 ± 12.7	75,1 ± 13.1	NS
FPG, mmol/l	5.65 ± 1.53	5.06 ± 0.65	< 0.001
Total cholesterol, mmol/l	5.45 ± 0.93	5.11 ± 1.12	< 0.01
LDL-C, mmol/l	3.55 ± 1.11	3.10 ± 1.62	< 0.01
HDL-C, mmol/l	1.42 ± 0.37	1.56 ± 0.37	< 0.05
Triglycerides, mmol/l	1.32 ± 0.74	0.95 ± 0.50	< 0.001
25(OH)D, nmol/l	46.62 ± 19.48	52.23 ± 19.93	< 0.05
**VITAMIN D STATUS**			
Deficiency, *n* (%)	342(65)	88(51)	NS
Insufficiency, *n* (%)	147(28)	61(36)	NS
Sufficiency, *n* (%)	37(7)	22(13)	NS

*Results are presented as means ± SDs and percentages. WC, waist circumference; BMI, body mass index; FPG, fasting plasma glucose; LDL-C, low density lipoprotein cholesterol; HDL-C, high density lipoprotein cholesterol; BP, blood pressure; 25(OH)D, 25-hydroxyvitamin D*.

Statistical analyses revealed an association between vitamin D deficiency and presence of abdominal obesity [CI (95%) 2.23; (1.15–4.30)] as well as low HDL-C [CI (95%) 2.60; (1.04–6.49)]. Serum 25(OH)D <39.0 nmol/l was associated with increased risk of IGT and T2DM [CI (95%) 7.17; (2.99–17.7)].

Metabolic syndrome components were evaluated in 397 women with abdominal obesity and genetic data. Among this group 187 subjects (47.1%) met IDF criteria of metabolic syndrome. Apart from abdominal obesity 187 subjects (47.1%) had increased plasma glucose or T2DM, 180 (45.3%) had reduced HDL-C, 152 (38.3%) were diagnosed with arterial hypertension, hypertriglyceridemia was revealed in 111 subjects (28.0%). The study showed that participants with MS had significantly higher systolic and diastolic blood pressure, fasting plasma glucose, LDL-C, TG, and lower levels of HDL-C compared to those with abdominal obesity but without MS. No significant differences were found for serum 25(OH)D concentration between the groups with and without MS. Vitamin D status did not influence risk of developing MS (*p* > 0.05). Clinical and laboratory characteristics of the subjects with and without MS are presented in Table 2.

**Table 2:** Characteristics of patients with and without metabolic syndrome.

**Characteristics**	**MS (+)**	**MS (−)**	***P*-value**
Age, years	45.4 ± 9.9	43.3 ± 5.1	<0.05
WC, cm	98.9 ± 11.1	87.6 ± 11.6	<0.01
BMI, kg/m^2^	31.5 ± 5.7	27.9 ± 5.1	<0.001
**BP, mmHg**			
Systolic	133.8 ± 18.7	121.17 ± 11.0	<0.001
Diastolic	85.9 ± 11.9	77.80 ± 11.7	<0.001
FPG, mmol/l	6.20 ± 1.78	5.30 ± 0.72	<0.01
Total cholesterol, mmol/l	5.60 ± 1.23	5.28 ± 0.87	<0.01
LDL-C, mmol/l	3.66 ± 1.23	3.34 ± 1.45	<0.05
HDL-C, mmol/l	1.21 ± 0.41	1.58 ± 0.43	<0.001
Triglycerides, mmol/l	1.70 ± 0.96	1.01 ± 0.29	<0.001
25(OH)D, nmol/l	48.62 ± 1.81	51.14 ± 1.56	NS

*Results are presented as means ± SDs and percentages. WC, waist circumference; BMI, body mass index; FPG, fasting plasma glucose, LDL-C, low density lipoprotein cholesterol; HDL-C, high density lipoprotein cholesterol; BP, blood pressure; 25(OH)D, 25-hydroxyvitamin D*.

Genotyping for rs1544410 (*BsmI*), rs7975232 (*ApaI*), rs731236 (*TaqI*), and rs2228570 (*FokI*) polymorphisms in VDR gene was performed in study subjects and in controls. Genotype and allele distribution did not differ between the groups. The observed genotype frequencies were consistent with Hardy-Weinberg equilibrium. Genotype distribution of VDR gene polymorphisms is shown in Table 3.

**Table 3:** Distribution of VDR genotypes in study subjects and controls.

**VDR genotypes (%)**	**Study population**	**Controls**	***P*-value**
**rs1544410 (*****BsmI*****)**			
GG *(BB)*	24.3	24.1	>0.05
GA *(Bb)*	57.7	54.2	
AA *(bb)*	18.0	21.7	
N	449	212	
**rs7975232 (*****ApaI*****)**			
TT *(AA)*	25.1	25.9	>0.05
TG *(Aa)*	54.9	51.5	
GG *(aa)*	20.0	22.6	
N	454	212	
**rs731236 (*****TaqI*****)**			
TT *(TT)*	45.1	45.3	>0.05
TC *(Tt)*	42.7	45.3	
CC *(tt)*	12.2	9.4	
N	454	212	
**rs2228570 (*****FokI*****)**			
CC *(FF)*	28.4	34.3	>0.05
CT *(Ff)*	50.5	51.3	
TT *(ff)*	21.1	14.4	
N	402	236	

There was no difference in serum 25(OH)D concentration and anthropometric characteristics between rs1544410 (*BsmI*), rs7975232 (*ApaI*), rs731236 (*TaqI*), and rs2228570 (*FokI*) genotypes. Laboratory results demonstrated significantly higher TG levels in subjects with GG (*BB)* genotype of rs1544410 (*BsmI*) polymorphism compared to A (*b*) allele carriers GA, AA (*Bb, bb*). Subjects with genotypes TT (*AA*) and TG (*Aa*) of rs7975232 (*ApaI*) polymorphism had significantly higher levels of TC and LDL-C compared to genotype GG (*aa)* carriers. No other differences between studied genotypes were observed (Table 4). Multiple regression analysis adjusted to age, smoking status, WC, BMI, and 25(OH)D level demonstrated no association of VDR gene polymorphisms with MS risk.

**Table 4:** Lipid profile, plasma glucose level and serum 25(OH)D concentration in relation to VDR genotypes.

**Genotypes**	**TC, mmol/l**	**LDL-C, mmol/l**	**HDL-C, mmol/l**	**TG, mmol/l**	**FPG, mmol/l**	**25(OH)D, nmol/l**
**rs1544410(*****BsmI*****)**						
GG *(BB)*	5.44 ± 0.14	3.44 ± 0.12	1.37 ± 0.04	1.54 ± 0.09^*^	5.66 ± 0.21	49.72 ± 2.57
GA *(Bb)*	5.37 ± 0.07	3.44 ± 0.06	1.39 ± 0.02	1.28 ± 0.04	5.59 ± 0.08	48.68 ± 1.96
AA *(bb)*	5.56 ± 0.13	3.60 ± 0.14	1.36 ± 0.10	1.40 ± 0.08^*^	5.72 ± 0.10	48.31 ± 3.18
*b allele carries*	5.40 ± 0.06	3.45 ± 0.06	1.38 ± 0.02	1.32 ± 0.04^**^	5.66 ± 0.07	50.05 ± 1.95
^*^p < 0.05 compared to *Bb* genotype, ^**^p < 0.05 compared to *BB* genotype						
**rs7975232 (*****ApaI*****)**						
TT *(AA)*	5.49 ± 0.15^#^	3.47 ± 0.13^#^	1.40 ± 0.05	1.46 ± 0.10	5.68 ± 0.13	54.39 ± 3.31
TG *(Aa)*	5.53 ± 0.08^#^	3.37 ± 0.07	1.37 ± 0.03	1.32 ± 0.05	5.80 ± 0.13	48.88 ± 2.42
GG *(aa)*	5.14 ± 0.15	3.25 ± 0.12	1.36 ± 0.05	1.40 ± 0.05	5.74 ± 0.14	49.86 ± 4.27
*A allele carries*	5.52 ± 0.07^#^	3.54 ± 0.06^#^	1.38 ± 0.02	1.36 ± 0.05	5.76 ± 0.10	51.11 ± 1.96
^#^*p* < 0.05 compared to *aa* genotype						
**rs731236 (*****TaqI*****)**						
TT *(TT)*	5.48 ± 0.08	3.53 ± 0.08	1.38 ± 0.03	1.35 ± 0.05	5.66 ± 0.09	48.29 ± 2.27
TC *(Tt)*	5.35 ± 0.07	3.39 ± 0.07	1.37 ± 0.03	1.31 ± 0.06	5.78 ± 0.13	49.88 ± 1.98
CC *(tt)*	5.61 ± 0.22	3.58 ± 0.19	1.36 ± 0.47	1.48 ± 0.13	5.59 ± 0.13	50.33 ± 3.60
**rs2228570 (*****FokI*****)**						
CC *(FF)*	5.42 ± 0.12	3.40 ± 0.06	1.40 ± 0.04	1.33 ± 0.07	5.60 ± 0.08	51.45 ± 4.21
CT *(Ff)*	5.47 ± 0.09	3.59 ± 0.08	1.34 ± 0.03	1.38 ± 0.05	5.71 ± 0.11	47.53 ± 3.56
TT *(ff)*	5.33 ± 0.11	3.41 ± 0.13	1.35 ± 0.21	1.33 ± 0.08	5.51 ± 0.7	45.90 ± 2.85

Results are presented as means ± SDs. VDR, vitamin D receptor gene; TC, total cholesterol; LDL-C, low density lipoprotein cholesterol; HDL-C, high density lipoprotein cholesterol; TG, triglycerides; FPG, fasting plasma glucose; 25(OH)D, 25-hydroxyvitamin D.

* compared to Bb genotype,

** compared to BB genotype,

# *compared to aa genotype*.

## Discussion

High prevalence of vitamin D deficiency is currently a global health problem (27). Low vitamin D status corresponding to either deficiency or insufficiency has been observed in all age groups from different countries and ethnicities (26, 28). According to our previous data, prevalence of vitamin D deficiency and insufficiency in northwestern region of Russia was observed in over 80% of the population studied (25), and was reconfirmed in this study. Such a high prevalence of low vitamin D status in middle-aged women is probably related to inadequate natural sunlight exposure, which is known to be a major cause of vitamin D deficiency (28). But what might be the impact of low vitamin D levels? The main physiologic function of vitamin D is maintenance of calcium and phosphate homeostasis and bone metabolism control but vitamin D effects are not just limited to bone tissue regulation as witnessed by the fact that most cells in the body have vitamin D receptors as well as para/autocrine vitamin D metabolic machinery (27, 29, 30). There have been an increasing number of investigations on the extraskeletal actions of vitamin D including regulation of cellular proliferation and differentiation, as well as effects on cardiovascular and immune systems (29, 30). Apart from musculoskeletal problems vitamin D deficiency has been found to affect a number of acute and chronic diseases (31). Recent studies have demonstrated an association between vitamin D deficiency and some metabolic disorders such as obesity, MS, and T2DM (32). As we know, MS is a combination of abdominal obesity and cardiovascular risk factors such as hyperglycemia, hypertension, and dyslipidemia associated with increased risk of cardiovascular disease (CVD). According to the IDF consensus abdominal obesity is defined as waist circumference ≥94 cm in males and ≥80 cm in females and is the main component of MS (6). Prevalence of abdominal obesity in our study was 75.5%. Patients with abdominal fat distribution had significantly higher levels of some metabolic parameters such as BP, FPG, TC, LDL-C, and TG and lower HDL-C related to CVD. Our findings appeared to be consistent with the previously reported data concerning association between central obesity and higher incidence of cardiovascular risk factors (33). Some studies have demonstrated an association between obesity and vitamin D deficiency but the causal relationship between these two conditions remains to be determined (34). In our study vitamin D deficiency was associated with increased risk of abdominal obesity and was noted more frequently in women who were overweight or obese. These results support previously demonstrated evidence of inverse association between 25(OH)D concentration and body mass index (35, 36).

Observational and prospective studies have shown the possible relationship between 25(OH)D concentrations and glucose levels (37). However, a recent study reported no association between 25(OH)D and risk of diabetes (38). According to data from systematic review and updated meta-analysis, 25(OH)D concentration was inversely associated with the incidence of T2DM (39). Our results did not show a relationship between glucose and 25(OH)D values in middle-aged women, however subjects with 25(OH)D levels less than 39 nmol/l had IGT or T2DM more frequently. We could not confirm that vitamin D deficiency played a role in increasing the risk of developing T2DM and hence a prospective study is required.

Furthermore, other studies have reported a link between vitamin D status and dyslipidemias and showed that low 25(OH)D concentrations were associated with increased TG (40, 41) and decreased HDL-C or apolipoprotein A1 levels (42). We found a positive association between 25(OH)D values and HDL-C, but no associations between 25(OH)D and total cholesterol, TG and LDL-C values. It is known that vitamin D and cholesterol share a common 7-dehydrocholesterol pathway and hence, it is possible that the relationship between 25(OH)D and dyslipidemias could be related to the synthesis of vitamin D precursor and lipoproteins in the liver.

Moreover, the effects of vitamin D are mediated by VDR that belongs to the steroid receptor family and modulates expression of target genes. VDR is a protein which consists of two functional domains (N-terminal dual zinc finger DNA binding domain and C-terminal ligand-binding activity domain) and linking region (43, 44). VDRs are widely expressed in different tissues. The gene encoding VDR is located on chromosome 12 (12q12-14) (45).

Several single nucleotide polymorphisms have been described in the VDR gene which are supposed to affect metabolic disorders related to vitamin D deficiency (46). Single nucleotide polymorphisms (SNPs), including rs1544410 (*BsmI*), rs7975232 (*ApaI*), and rs731236 (*TaqI*), located at the 3′ untranslated region of VDR gene have been shown to influence mRNA stability and VDR expression (22, 46) whereas rs2228570 (*FokI*) SNP located near the promoter region results in altered VDR activity due to change in amino acid sequence of this protein (47). Genetic variants in VDR gene have been reported to be associated with MS and its components including anthropometric parameters related to obesity, insulin resistance, T2DM, and atherogenic lipid abnormalities in different populations (21, 23, 48). In contrast to these data, some studies have demonstrated no association between VDR gene polymorphisms and the risk for MS development (49–51). Our study found no significant relationship between VDR SNPs and serum 25(OH)D concentration, anthropometric characteristics, FPG, and MS risk, however an associations of rs1544410 (*BsmI*) and rs7975232 (*ApaI*) variants with atherogenic lipid profile were revealed. Thus, GG (*BB)* carriers of rs1544410 (*BsmI)* showed significantly higher TG levels, whereas, women carrying TT (*AA)* and TG (*Aa)* genotypes of rs7975232 (*ApaI)* had significant increase in TC and LDL-C. Consistent with our results, some previously reported studies showed no association between VDR gene polymorphisms and MS risk but observed the relationship of VDR SNPs with dyslipidemia (49, 51).

## Conclusion

Our study demonstrated very high prevalence of low vitamin D status in middle-aged women from North-West Russia. We found an association between vitamin D deficiency and increased risk of MS components such as abdominal obesity, reduced HDL-C, IGT, and T2DM. VDR gene polymorphisms rs1544410 (*BsmI*), rs7975232 (*ApaI*), rs731236 (*TaqI*), and rs2228570 (*FokI*) were not associated with MS risk in our study but the relationship between rs1544410 (*BsmI*) variants with TG levels and rs7975232 (*ApaI)* variants with TC and LDL-C was identified. Our findings suggest that vitamin D deficiency and VDR gene polymorphisms may contribute to the development of some MS components in adult female population. Future studies are required to clarify the causal relationship between VDR gene polymorphism, low vitamin D status and metabolic disorders including developing T2DM.

## Ethics statement

This study was carried out in accordance with the recommendations of Ethics committee of Pavlov First Saint Petersburg State Medical University. The protocol was approved by the ethics committee of Pavlov First Saint Petersburg State Medical University. All subjects gave written informed consent in accordance with the Declaration of Helsinki.

## Author contributions

TK designed the study, performed the research, wrote the paper. EG and OB designed the study, performed the research. AB, AA, and AK performed the research, wrote the paper. EJ and PP wrote the paper.

### Conflict of interest statement

The authors declare that the research was conducted in the absence of any commercial or financial relationships that could be construed as a potential conflict of interest. The handling Editor and author PP declared their involvement as co-editors in the Research Topic, and confirm the absence of any other collaboration.
